# Staying ‘in the zone’ but not passing the ‘point of no return’: embodiment, gender and drinking in mid-life

**DOI:** 10.1111/1467-9566.12103

**Published:** 2014-01-22

**Authors:** Antonia C Lyons, Carol Emslie, Kate Hunt

**Affiliations:** 1 School of Psychology, Massey UniversityNew Zealand; 2 Institute for Applied Health Research, School of Health & Life Sciences, Glasgow Caledonian UniversityScotland; 3 Medical Research Council/Chief Scientist Office, Social and Public Health Sciences Unit, Glasgow UniversityScotland

**Keywords:** alcohol consumption, embodiment, gender, health behaviour

## Abstract

Public health approaches have frequently conceptualised alcohol consumption as an individual behaviour resulting from rational choice. We argue that drinking alcohol needs to be understood as an embodied social practice embedded in gendered social relationships and environments. We draw on data from 14 focus groups with pre-existing groups of friends and work colleagues in which men and women in mid-life discussed their drinking behaviour. Analysis demonstrated that drinking alcohol marked a transitory time and space that altered both women's and men's subjective embodied experience of everyday gendered roles and responsibilities. The participants positioned themselves as experienced drinkers who, through accumulated knowledge of their own physical bodies, could achieve enjoyable bodily sensations by reaching a desired level of intoxication (being in the zone). These mid-life adults, particularly women, discussed knowing when they were approaching their limit and needed to stop drinking. Experiential and gendered embodied knowledge was more important in regulating consumption than health promotion advice. These findings foreground the relational and gendered nature of drinking and reinforce the need to critically interrogate the concept of alcohol consumption as a simple health behaviour. Broader theorising around notions of gendered embodiment may be helpful for more sophisticated conceptualisations of health practices.

## Introduction

Alcohol consumption and excessive drinking have received considerable research and public health attention. Much of this work has conceptualised alcohol consumption as an individual behaviour resulting from rational choice, although public health approaches based on such assumptions are relatively ineffective (Babor *et al*. 2010). Some researchers have explored drinking behaviour in its social context, employing in-depth qualitative methodologies to theorise drinking as a practice located in people's social worlds (for example, Griffin *et al*. 2009, Lyons and Willott 2008). This research has conceptualised heavy (binge) drinking behaviour as calculated hedonism, a controlled loss of control (Measham 2006) and a way of signalling taste and identity preferences (for example, McCreanor *et al*. 2005). Yet it has focused almost exclusively on young people and paid little attention to drinking practices in terms of embodiment. Here we explore how men and women in mid-life represent their alcohol consumption as an embodied social practice. Embodiment is central to drinking practices as it allows the consideration of emotions, feelings and gender, highlights the complexities of drinking behaviour and emphasises the limitations of individual-level approaches to health practices.

Individually focused health promotion efforts to reduce alcohol consumption fail to capture the meanings and the context of drinking. Interventions frequently focus on increasing people's knowledge of a particular behaviour (for example, a recommended number of standard alcohol units) and assume that people will automatically amend their drinking in line with recommendations. This social cognition approach has been heavily criticised for its lack of success in predicting or changing behaviour (Mielewczyk and Willig 2007), being simplistic (Richmond, 1998) and conceptually problematic (Ogden 2003), portraying individuals as primarily rational beings whose behaviour is devoid of social context or social meaning (Backett and Davison, 1995) and failing to take affective factors into account (Mielewczyk and Willig 2007).

While physiological and behavioural responses to alcohol intake are most easily understood as phenomena occurring within an individual body, we argue that how these are experienced and interpreted is inevitably social, cultural and gendered. Choosing to drink alcohol, and decisions about continuing to drink, take place within our experience of both having and being a gendered physical body. Most young adults strive to keep some control over achieving and maintaining a desired state of intoxication, carefully choosing what, when and where to drink, who to drink with and when to stop or slow drinking (Measham 2006). These decisions and their meanings are intertwined with cultural practices of gender (for example, Griffin *et al*. 2013, Willott and Lyons 2012). Yet we know little about the processes through which such controlled intoxication is achieved and maintained, what people's gendered embodied experiences are and how they influence such decisions. We know even less about older men's and women's embodied experiences of drinking or of exceeding acceptable levels of intoxication and their decisions to cease drinking alcohol during a specific drinking episode. There are material limits to consuming alcohol within a drinking session due to the consequences of alcohol on physical bodies (for example, slurring, passing out and vomiting). Exploring these material limits, and how they are understood by men and women from different backgrounds and life stages, is crucial to understanding drinking practices. Furthermore, conceptualising drinking as an embodied practice allows fuller consideration of emotions and feelings such as pleasure, which is corporeal and subjective, felt and experienced within the body (Duff 2008). Health-promotion approaches aiming to reduce consumption tend to privilege cognitive and perceptual factors over these pleasurable, corporeal experiences (Duff 2008).

Scholars have argued for a consideration of gendered embodiment in both alcohol research (for example, Thurnell-Read 2011) and critical perspectives on health promotion and public health (Robertson and Williams 2010). Drinking is a gendered activity, with men drinking more often and more heavily than women internationally (Rahav *et al*. 2006), and traditionally expected to drink (primarily beer) excessively and in public (Lemle and Mishkind 1989). We enact varied gender identities by taking part in behaviour that has cultural meanings that are associated with versions of masculinity and femininity (Hunt *et al*. 2013, Lyons 2009); here gender is an ongoing bodily performance (Butler 1993). Young men and women engage in particular drinking practices to perform and maintain desired gender identities (de Visser and McDonnell 2012, Lyons and Willott 2008). Men use public drinking to demonstrate their relationship to hegemonic masculinity (Peralta 2007, Willott and Lyons 2012), a set of idealised social practices and norms that legitimise the interests of the powerful and the dominant position of men over women (Connell 1995). Simultaneously, young women limit or control their drinking, being aware that breaching traditional feminine boundaries may lead to their being seen as bad, promiscuous or masculine (Griffin *et al*. 2013, Peralta 2007). Older women talk about their drinking in ways that resist and deflect the stigma often associated with excessive drinking in women (Rolfe *et al*. 2009), illustrating how moral discourses around drinking are highly gendered (Day *et al*. 2004). Men and women drink heavily for pleasure and fun but this plays out in different ways for masculinities, femininities and gender relations for young (Lyons 2009) and older adults (Emslie *et al*. 2012, 2013).

While informative for linking drinking practices with gender identities, this work rarely considers the body. Yet the corporeal body is crucially important, as it is in and through our bodies that we negotiate daily life and experience the world. Young women's drinking practices in rural England have been shown to be embodied performances negotiated in relation to rural space and society (Leyshon 2008). Thurnell-Read's (2011) research with men drinking on a stag tour in Eastern Europe found they displayed an unconstrained embodiment (in contrast to traditional bounded and controlled embodied masculinity), which involved pleasurable bodily transgressions, the display of leaky bodies (through vomiting and urination) and illness and fatigue. He argues that ‘the unruly drunkenness of collective drinking rituals has become, for many men, the location of their most notable or, at least, most vivid engagement with their own bodies’ (p. 987) which functions to reassert male friendships and bonds.

Our research was designed to address the call for a ‘richer and fuller understanding of the relationship between embodiment, emotions and alcohol, drinking and drinking practices’ (Leyshon 2008: 285) and provide further knowledge of men's and women's drinking in mid-life. Below we utilise empirical data to argue that alcohol consumption needs to be understood as an embodied social practice that occurs at the complex intersection of the physiological effects of alcohol, embodied gendered identities, emotions and feeling states, and external materialities including places, people and spaces.

## Study background, design and method

The DrAM (Drinking Attitudes in Mid-life) study aimed to explore experiences and understandings of alcohol consumption in mid-life adults (see also Emslie *et al*. 2012,2013 ). We defined mid-life as ranging from 30–50 years to distinguish our participants from the younger adults who are frequently the focus of alcohol research. The study was informed by a social constructionist epistemology, which views the world as involving multiple systems of understanding that occur through social and cultural experiences, which in turn are largely influenced by the active and constructive nature of language (Burr 2003). This raises some difficulties for studying embodiment, as social constructionism has no adequate notion of embodied subjectivity (Cromby 2004) and lived bodily experience is not always reducible to language. We drew on Cromby's (2004) notion of embodied subjectivity to develop an integrated critical realist constructionism that takes both materiality and embodiment into account. Here the physical, material body and its corporeal processes are recognised but it is acknowledged that their meanings are socially constructed, subject to change culturally and historically. As Robertson and Williams (2010: 59) argue, ‘the representational and the material aspects of bodies are not readily separable’; our understandings of bodies and being in the world are wrapped up in discourse. Thus, we sought to explore embodied experience by asking people explicitly to recall their lived embodied sensations, feelings and states when they consumed alcohol. As we were also interested in the inherently social nature of drinking and how these subjective experiences were interwoven with the social, we conducted discussion groups with people who were friends or work colleagues and who drank together occasionally. We chose to conduct same-sex and mixed-sex groups to provide greater diversity in the contexts of the discussions, and to explore similarities and differences in gendered, embodied experiences across different groups.

Our recruitment strategies involved approaching potential participants on the street and in bars, e-mailing people, inviting them to forward study information to friends and colleagues, contacting community groups and workplaces and advertising on community websites. People interested in taking part were asked to invite up to five friends or colleagues in the desired age range who regularly drank alcohol to join them in a group discussion with a researcher. The participants gave their written, informed consent to be audiotaped and completed a drinking grid estimating their alcohol consumption in the previous week. Discussions were facilitated by CE using a semi-structured interview schedule covering topics such as changes in drinking over time, occasions and feelings when participants had drunk more than they intended, attempts to reduce drinking and distinctions between men's and women's drinking. The participants were given a £20 voucher towards compensating them for their time and costs incurred. Group discussions lasted between 60–95 minutes, were transcribed verbatim and checked against the recordings for accuracy. Pseudonyms were used and any identifying features were changed or removed. Detailed field notes were written after each focus group and shared with the research team.

In all, 15 group discussions were conducted in Glasgow between 2009 and 2011 following approval from Glasgow University's Faculty of Law, Business and Social Sciences Ethics Committee. Here we present data from 14 groups (six mixed sex, three all-male and five all-female), excluding a group of non-drinkers (FG7) recruited to provide a different perspective on the cultural context of alcohol. Details of the groups are provided in Table 1. The 56 participants (22 men and 34 women) were all white, aged mostly in their thirties and forties and came from diverse socioeconomic backgrounds. A total of 30 participants lived with a partner and 25 had at least one child under 18 years of age living with them on a day-to-day basis. The participants reported having consumed a range of units of alcohol in the previous week (where each unit represents 8 grams of pure alcohol). Two participants had not consumed alcohol, while 16 men and 16 women had consumed in excess of the threshold for hazardous drinking (over 21 units of alcohol for men and 14 units for women) according to UK National Health Service (2011) guidelines.

**Table 1:** Details of focus groups and participants (*N* = 56)^1^

Group type	Participants	Ages	Deprivation category^2^	Alcohol units past week	No. drinking at ‘hazardous’ level^3^
FG1 Council workers	2 M, 2 F	44–49	intermediate	9–15	1 F
FG2 Male pub friends	4 M	44–50	mixed	49–90	4 M
FG3 Lecturers	2 M, 2 F	34–49	mixed	21–33	2 M, 2 F
FG4 Female friends	4 F	44–48	affluent/ intermediate	14–60	3 F
FG5 Sales workers	1 M, 4 F	40–50	mixed	9–36	1 M, 3 F
FG6 Community group (deprived area)	4 M, 3 F	41-mid 50s?^4^	deprived	0–92	1 M
FG8 Office workers	4 F	36–47	affluent/ intermediate	0–27	2 F
FG9 Community group (affluent area)	3 F	35–45	affluent	3–15	1 F
FG10 Heterosexual couples	2 M, 2 F	32–35	affluent/ intermediate	14–28	1 M, 1 F
FG11 Best friends and girlfriend	2 M, 1 F	31–33	mixed	25–65	2 M, 1 F
FG12 Gym group mums	4 F	30–31	deprived	3–20	1 F
FG13 Unemployed friends	2 M	28–31	affluent/ intermediate	29–38	2 M
FG14 Old school friends	3 M	30–32	deprived	27–48	3 M
FG15 Toddler group mums	5 F	30–41	affluent/ intermediate	1–19	1 F

1 This dataset excludes group 7, a group of non-drinkers recruited for an alternative perspective on the cultural context of alcohol.

2 Carstairs scores calculated for residential postcodes: affluent = DEPCAT 1 and 2, intermediate = DEPCAT 3–5, deprived = DEPCAT 6 and 7, mixed = respondents from each of these three categories present in one group.

3 ‘Hazardous’ drinking: more than 21 units/week for men, 14 units/week for women.

4 Two participants in FG6 did not give their age.

F, female; M, male.

Thematic analysis (Braun and Clarke 2006) allowed us to identify, analyse and report patterns in the data, and thus provide rich, detailed and complex accounts informed by our theoretical frameworks. The transcripts were read a number of times by all authors. One researcher (AL) identified initial patterns in the data, which were then coded for more detailed scrutiny. This process generated 12 general themes and sub-themes (for example, feelings-emotions, feeling-body states, physiology, contextual factors, reasons to drink/stop drinking, social experiences, ageing and health-promotion messages). Another author (CE) independently coded the transcripts, focusing on gender and gender roles. There was a significant overlap in the themes identified, particularly around reasons given for drinking, feelings and emotions and physiological responses to alcohol. Next AL reformulated these initial themes into higher order conceptual groupings, focusing primarily on bodies, including embodied pleasures, constraints and experiences. These themes were reworked repeatedly through an ongoing discussion with all researchers to ensure they were grounded in the data and oriented to gender.

## Findings

The participants varied widely in how much alcohol they consumed, and how often, where and what they drank. Most distinguished between their drinking practices when they were younger, which were associated with the physical effect of alcohol on the body and getting drunk, and their current, wiser, drinking practices which they framed as being more about enjoyment, and taste (see Emslie, Hunt, and Lyons 2012). Yet the participants also recounted stories of recent drunken or heavy drinking episodes and social pressures to drink. Despite this diversity of drinking practices there were striking commonalities in the data around the pleasurable sensations that alcohol and drinking practices provided, the point at which participants just knew it was time to curtail or cease drinking, the factors that influenced their reducing consumption and their awareness of public health information. Below we highlight these key similarities in relation to gendered material bodies.

### Embodied pleasures of drinking practices and alcohol consumption: older bodies and the material world

The participants all described their enjoyment of alcohol. This was most commonly associated with feelings of relaxing, winding down, stopping activity and distinguishing between everyday practices and rest time. It was discussed as making ‘you feel nice and calm’ (Cath FG4), as taking away ‘the aches and pains from the day's work’ (Craig FG1), and providing that ‘nice sense of relaxation’ (Anne FG12). Alcohol consumption also marked a space away from domesticity and childcare for many women; for example Anne (FG12) framed drinking as ‘a declaration of adulthood’ and Madeleine (FG15) as ‘the end of Mummy day’.

Below Michael explains how the immediate embodied sensation of drinking a chilled glass of wine at the end of the day evokes a strong, positive emotional response:

Michael: Wine's a good comforter – you know? If you've had a hard day at work or something. I used to work quite, I won't say stressful but it was relatively stressful at one time, you know? And you used to come in and get a glass of wine just tae [to], you know?

Grace: Oh yes, yes, I've had that.

Michael: The cold hit at the back of the throat and you think, ‘Oh, that's my best pal’. [laughter] (FG5)

Hugh provided a vivid illustration of the embodied enjoyment of anticipating going to the pub and drinking with his mates, and constructed drinking in traditional embodied masculinity terms, involving working hard, then having the reward of drinking at the pub:

Hugh: You know, the thing is that, going to the pub feels to me like how I used to feel when I was 10 and I wanted to go out to play. It's that feeling of, ‘Right, I can cope with school, I can cope with doing all the boring stuff, blah de blah de blah’, but you're just *bursting* to get out with your mates … You know most of the time, you know you've got at least a 35-hour working week, maybe more, you know. And, week in, week out, month in, month out. And you have to have the discipline to get through all that, whatever it is you're doing, *grind* most of the time … it is like a reward. (FG2)

Some women also discussed alcohol enabling an escape from the grind of mundane work, but in the different context of unpaid domestic work or combining domestic and paid work, reinforcing traditional notions of femininity involving the domestic realm:

Hannah: We always have wine with our meal because, you know, I like to set the table and sit down and, you know, ‘coz it's the only chance you get to sit down and have a, you know, an hour sort of niceness, then, before you've got to load the dishwasher, do the ironing, you know, and so on and so forth. (FG8)

In contrast, some participants said they did not drink alcohol to relax or escape, but to binge drink and get drunk, which Ruth described as ‘a release valve’:

Ruth: It's not really about relaxation for me, as such –

Lynn: Yeah, I'm the same –

Ruth: It's, like, about sociable, party –

Lynn: I think, yeah, I think – I drink to get drunk. I think, unless I'm with people who aren't like that, and I would have a glass of wine not thinking – no, but I think I do, I do think I'm definitely a binge drinker … I don't think I could ever just be a moderate drinker. It's – you binge. You don't do it again for 3 months. You binge. And it's just like that, you know.

Ruth: It's like a kind of, like, release valve, like a kind of pressure valve, letting off steam. (FG12)

Here Ruth and Lynn describe a particular embodied engagement with their social world, where feelings of pressure built over time were released through binge drinking. The pressure valve metaphor implies catastrophe if release is not achieved, representing drinking as both necessary and important and intimately tied to experiential, embodied contexts.

Drinking alcohol marked a transitory time and space that altered participants' subjective embodied experience to differentiate it from normal, everyday embodied experience (see Jayne *et al*. 2010, Leyshon 2008). This was gendered due to the differences in everyday social roles (such as childcare, paid and unpaid work and housework). Generally, for men, drinking alcohol provided embodied pleasure as a reward for working hard, while for women it enabled an embodied sense of enjoyment, winding down and escape from busy lives involving multiple roles.

### Mid-life bodies: constraints to drinking

Both the materiality of ageing bodies (thought to process alcohol less well than youthful bodies, leading to unpleasant hangovers) and the social contexts of being in mid-life (such as the responsibilities of paid work and childcare) were perceived to limit excessive drinking. Participants found it difficult to fully disentangle the effects of ageing from social constraints, as Vicky described in relation to her body not ‘handling it as much now’:

Vicky: The headaches last for longer and they're probably a bit more intense. You find, as well, that you can't lie in bed all day the day after – you've got things to do that you didn't have to do before – so you're kind of dragging yourself around. You're just punished for it a lot more. I think that really is a physicality of just getting older. So that means that you're less likely to binge. (FG6)

Ageing bodies and social responsibilities were the primary reasons given for reducing alcohol consumption. Decisions to moderate alcohol consumption were also influenced (to a lesser extent) by driving the following day and weight concerns (see Emslie, Hunt, and Lyons 2012). Two men and two women mentioned weight considerations when deciding whether and what to drink, with Fi (FG10) commenting that if she wanted to lose weight ‘I'll consciously think, right, I'm not gonnae [going to] drink or I'm gonnae drink a spirit instead of a wine’ and Anne (FG12) saying ‘I've just begun to think I would much rather have that many calories in a chocolate rather than in beer form!’

Many participants discussed gender differences in size, physiology and hormones in relation to tolerance to alcohol and the different health-promotion advice given to men and women about drinking. Audrey's account illustrates her embodied experience of her changing tolerance to the physiological effects of alcohol, which she linked to hormonal changes:

Audrey: It is like a small poison. I mean, that sounds ridiculous, but if it hits me at the wrong time of day and, probably the wrong time of the month as well, and maybe even when I've got other things on my mind – all these things combined, then it tastes like it is something that my body is *rejecting* … It's physical. It's hugely physical. (FG8)

The participants were aware of public health alcohol advice and recommendations regarding units, which have been central to public health and prevention approaches. However while this knowledge increased their thinking about their health outcomes, it did not alter their drinking practices. Some were quite cynical about health promotion advice on alcohol and units, which was viewed as shifting sands (Stella FG15), arbitrary (Madeleine FG15) and a bit abstract (Elizabeth FG9). Thus, when limiting their consumption participants did not count units (see also Jayne *et al*. (2012), but instead monitored their changing embodied states.

### Embodied knowing: staying in the zone but not passing the point of no return

The feelings attached to social drinking practices were frequently concerned with going with the flow and enjoying the good atmosphere and mood derived from alcohol, company and environment, as Matt explains:

Matt: It's spontaneous now, if I ever sort of get carried away, and drink a lot, it's because it's been spontaneous, you're in good company, you're having a good night, you're really enjoying it, you're enjoying the company, you're enjoying the chat, whatever, and you just keep going. (FG10)

These positive feelings were described consistently as loss of inhibitions, exaggerated responses to everything, becoming ‘an enhanced person – 10 per cent more charismatic, 10 per cent more exciting, 10 per cent more funny’ (Callum FG13). Both men and women described this state of enjoyment as being in the zone or that perfect level:

Bill: I'd love to drink *all* night but not get drunk. Get to that level of – that perfect level, you know, and stay there … in the zone, that's what it is.

Facilitator: Yeah. So is the zone, is that kind of feeling it a little bit then? Is that –

Bill: [Overtalking] Uh huh, that's when you've managed to untie your tongue enough and your brain seems to be working faster, and everything you say is really fascinating to other people [laughs] – and that happens to me all the time! But it's for about 10 minutes or something [laughter]. And thereafter it's all downhill. But there is that wee point where you think, I'm really enjoying myself. [Others agree]. And then there's the point where you get – the next day you're probably thinking, ‘And that's when I should have stopped’.

Eleanor: I know.

Facilitator: And is that alcohol or is that people, or is it both?

Bill: I think it's just both. It's the reduction of inhibitions so that you're feeling more confident and cheerful perhaps. It's the social context as well. (FG3)

Here the desirable feeling arises from the interplay between the physiological effects of alcohol and the social context. A key part of achieving this perfect level was sharing this with people who were ideally getting in the zone or on the same wavelength (Eric FG10). The social bonding over this calibrated consumption could mean that being the only sober person was ‘horrible’, ‘just terrible’ and socially isolating.

This desired embodied state was also influenced by participants' moods, frame of mind, environment, time of day, the stage of women's hormonal cycle, what alcohol was being consumed and how much food had been eaten. Alongside achieving this enjoyable state, the participants talked about reaching a tipping point or a point of no return (Mandy FG10, Callum FG13), when they realised they had consumed too much alcohol and were feeling less pleasant sensations. Women were much more likely to describe acting on this knowing sensation by stopping drinking:

Mandy: You just start to get a sensation that, maybe, I really don't know, maybe things start to slow down a tiny bit, or maybe you start to realise your own behaviour is, like, maybe you're, you know, you spill something, or you, like, make a mistake when you're speaking, like you say the wrong word, or something like that, and you think, ‘That's not normal for me’; I'm at this, you know, I've sort of gone beyond the stage of, you know, what I'm happy with. But it's quite, it's very hard to articulate the actual physical sensation of it, I think. (FG10)

Tara: I feel like I've developed an internal kind of gate, which I know I don't want to go through – and I don't really know what it is, I just *know* there's been quite a lot of nights out where I've just gotten to the point where I'm like, ‘If I have one more drink, I'm really not going to be happy. It's just gonna push past that point, and I just know that I don't want anymore’, and I'll just stop … And it must be, through many years of experience of me [laughs] going past that point – and I think it's hard to verbalise the feeling that it is. I mean, it's not necessarily that I feel sick or I feel dizzy – although, they would be obviously strong signs. It's, there might be a point of being full – of just being full and, I don't know, maybe happy. I don't know, I'm just like, ‘No, that's it. That's enough’. It's definitely subconscious, I think. (FG11)

Tara's embodied feeling of knowing when to stop consuming alcohol (her internal gate) developed through experience and thus now operated at a ‘subconscious’ level, implying that bodily experiences become so well-rehearsed that they no longer require conscious intervention or scrutiny. Some women also linked getting to the point that they have had too much with ‘thinking about the next day. Your time's too precious’ (Isobel FG8). While the women were willing to engage with questions around how they knew when they had drunk enough, many men noted that they just know. For example, Paul (FG6) talked about how ‘Something tells me I've had enough … My brain, I'm just trained, I just know when I've had enough’ Graham (FG2) described it as ‘just an internal thing you just know’, while Ewan (FG2) stated that ‘instinct is experience coalesced’.

The corporeal feelings of going past one's limit were described in various physical and embodied ways (and appeared easier for participants to articulate than describing approaching that limit). For example, participants talked about not being able to communicate, stumbling, slurring, having double vision, an upset stomach, numb gums; feeling nauseous, queasy, fuzzy, blurry, tired; keeling to the side, bumping into people and becoming too loud. They also described it as when ‘the liquid won't go down their throat’ anymore, feeling bloated, and getting to a point where they no longer liked the taste of alcohol. In three groups, participants discussed blackouts (FG5, FG13, FG14); for Finn (FG13), it was ‘passing out in the club, done that once’ while for Rach (FG5) blackouts occurred ‘all the time’:

Rach: I get blackouts wae [with] wine all the time – get blackouts, just don't remember. [laughs] … So now I don't drink wine when I go out. I very, very rarely, don't I? Very, very rarely.

Grace: No, you're on the vodka maybe more …

Facilitator: So you can know where you are a bit more?

Rach: Aye, because I know that once I go on that wine, I just don't remember. And I do, literally, take blackouts. I just don't remember – get up in the morning and all my lights is on, my telly's on, the fire's on. (FG5)

Gavin and Mark discussed drinking to a point that they were not able to remember what happened and Mark described having ‘that blackout at the end of the night’, implying these were a relatively regular (and normative) experience:

Gavin: See my problem is I don't know, I just wake up the next day and go, ‘Right, well, I don't remember, from this point on, so I must've but’ – so that's essentially my problem. But then yeah.

Mark: You can probably tell we know each other quite well, but we've discussed this before, it generally tends to be more when we're drinking whisky that you have that blackout at the end of the night. [Others agree] How did I get home, sort of thing. (FG14)

Both male and female participants discussed vomiting after excessive alcohol consumption, as illustrated by Susan below. In the next extract Callum explains how he would preplan what he ate before a ‘fairly big night out’ in anticipation of being sick from excessive drinking:

Susan: I can have a bottle of wine and I really, I'm OK, I've obviously been drinking but I'm OK. If I have a bottle and a *glass* then something happens,

Cath: It's that glass that did it! Not the bottle, it's the glass.

Susan: I turn my head to the side and it's such a queasy feeling that I am actually, I'm sick, so I know that that's me, I'm absolutely finished. [Others agree] And it does make me feel really nauseous or else I *am* physically sick and I can't have any more. That's that. (FG4)

Callum: Subconsciously as well, I would *always*, if I knew I was going out for a fairly big night out, have soup. This sounds really, really grim, like it sounds terrible, but what I'm thinking is, ‘What if I get so drunk that I'm sick, or even sick in my sleep. If I'm being sick liquid, I'll survive’. [Laughter]. But I think if I have a big meal, I'm far more likely to be sick, and also if I'm sick and it's just soup at least it's liquid coming out, if it's horrible big, you know, meat or whatever, then it's going to be fatal. And you shouldn't be thinking like that, but [overtalk] that's always in there. (FG13)

Lynn and Ruth (FG12) discussed making themselves vomit so they could keep drinking during a night out. Ruth was reluctant to discuss this topic, while Lynn appealed to their friend Anne not to ‘judge’ them, illustrating their awareness of the gendered morality that is attached to drinking (Day, Gough, and McFadden 2004), particularly behaviour linked to excessive consumption.

Changing physical stance and/or moving away from the immediate social situation (for example, going to the toilet or standing up) was frequently the first moment when participants realised that they had passed that limit. These social and physical perturbations act as sobering moments; they provide time out for a more objective assessment of physical state. Jeff (FG6) commented that ‘it doesn't really hit me till I get out in the air’, while Jody (FG5) noted ‘I just feel perfectly fine and then I'll go to stand up, or go to go into the kitchen or something’. Group 12 described these moments as follows:

Erin: Yeah, it's the going to the toilet thing, and realising that it's [alcohol] having an effect. ‘Coz I think – it's more like you're either talking or you're just like, busy, and then you're – going to the toilet's the first time you actually get to think how the night's going and then, ‘how many drinks have I had’. [Group laughter].

Ruth: I hadn't actually thought about it like that but it probably is.

Erin: It's the first time you're kind of like – the lights are different and you're like oh!

Lynn: The music isn't in your ears (FG12)

Thus the embodied, subjective experience of intoxication involves physical sensations in the body, but their interpretation depends upon the context, environment, space and place in which people are located. Alcohol facilitates togetherness and sociability, and drinking with others in the same space enables shared embodied experiences with everyone aiming for an ideal level of intoxication. Knowing when to stop and heeding the physical signs that are hard to articulate was linked to age, gender and experience. Women were more willing to state that particular physical sensations led them to stop drinking, quite suddenly at times. Men may present themselves as drinking heavily without a great deal of consideration, given that this behaviour is itself part of hegemonic masculinity. In contrast, women may take more notice of bodily signs and sensations. Femininity has traditionally been linked to an awareness of health and the body, thus women may be more conscious of physical changes while drinking, perhaps finding it easier to describe feelings of intoxication and bodily sensations than men. Further, women may monitor such changes and cease drinking when they occur because hegemonic femininity requires women to remain in control (ensuring respectability). However for both women and men, their own experiential and embodied knowledge was more important in regulating consumption during a drinking episode than health promotion advice.

## Conclusions

This research demonstrates that drinking is an embodied social practice that is both gendered and related to age and life stage. For these adults in mid-life, drinking with others was about marking out temporary spaces from everyday life in which they could alter their way of being in the world. This was frequently gendered; a shift from domestic work and childcare (often in addition to paid work) for women, and from paid work for men. Furthermore, by mid-life, participants were able to draw on a history of drinking over at least a decade or two that enabled them to position themselves as ‘experienced’ drinkers who knew their own physical bodies, how to achieve a desired level of intoxication and how to sustain this level by knowing when they had passed the point of no return and needed to stop drinking. Embodied feelings or sensations of intoxication, and decisions about when to stop or moderate their drinking, were articulated in more detail by women than men.

These findings foreground the relational and contextual nature of drinking practices and reinforce the need to critically interrogate the concept of alcohol consumption as a straightforward health behaviour. Most health behaviour theories exist at an individual level and neglect the contextual realities and broad influences that shape behaviour (Crosby and Noar 2010). Our results demonstrate that health promotion information is competing with, and undermined by, embodied knowledge accumulated through years of experience of drinking. The enjoyable, embodied and affective nature of drinking practices experienced by men and women at mid-life are in stark contrast to current political, policy and popular concerns regarding alcohol (Jayne, Valentine, and Holloway 2010). As Griffin *et al*. (2009) have argued, excessive alcohol consumption is positioned in governmental discourses as irresponsible, risky and dangerous and as ‘away from the rationality, self-control and moderation that is at the heart of neo-liberal subjectivity’ (p. 460). The adults in our study described end-of-day controlled drinking as a rational activity within the context of their busy lives and, for women, their multiple roles. They also employed notions of experience and embodied knowing while drinking to resist being positioned as irresponsible or risky drinkers.

If individualised health behaviour messages are ineffective for men and women in mid-life, what is required to alter potentially harmful drinking practices? Our findings highlight drinking as a collective activity involving the achievement of shared desirable embodied states and the importance of not going past the point of this enjoyable embodied intoxication. Health promotion approaches may usefully draw on these notions of embodied pleasure while drinking and embodied knowing of excessive drinking. Physical perturbations (for example, standing up or going to the toilet) can act as sobering moments providing a space to (re)assess one's physical state and decide to stop or slow down drinking, and may be key points for intervention strategies to focus on. Future research might usefully focus on alterations in environmental contexts and the cues that may facilitate this process.

The lived meaning of embodied drinking practices cannot be divorced from gender. Women's drinking was more strongly associated with their emotional and relational lives and men's with their external work lives, highlighting gendered styles of engagement with alcohol (Ettorre 2004). As gendered bodies consume alcohol for pleasure and enjoyment, they appear to be monitored and controlled more closely by women than men. In regulating consumption, ‘bodies are bound up in society's values of discipline and order, essential to wellbeing’ (Ettorre 2004: 331); bodies are the site for performing masculinity and femininity, as well as the site for regulation (Gill *et al*. 2005). Drinking for pleasure may be a resistance to gender roles, caretaking or passive femininity (Ettorre 2004). Simultaneously, alcohol was a way for both men and women to cope with the different demands and responsibilities of their daily lives. It is notable that the alcohol industry has succeeded in aligning drinking with reward and relaxation for daily coping among men and women at mid-life, as well as with celebrations and special occasions.

Our exploratory findings need to be considered cautiously. We employed friendship discussion groups to investigate both subjective embodied experience and co-constructed accounts of socialising and drinking together. This may have encouraged discussion of embodied experiences and heightened the level of similarity of the topics and ideas discussed but it may also have limited the disclosure of more personal, private or sensitive material on individual embodied experience. Future research could beneficially explore the use of individual interviews or ethnographic approaches to gain further insights.

Embodied, gendered experiences are central to drinking practices and are important in conceptualising health behaviour as beyond the individual. We cannot fully comprehend drinking and alcohol consumption practices without the consideration of bodies and their social and cultural location in terms of age, gender and life stage. Health promotion needs to move beyond this neglect of the body and engage with drinking as a gendered, embodied activity. Bodies are intertwined with self and culture and inextricably link the material and the discursive (Gill, Henwood, and McLean 2005). Our research suggests that the drinking, mid-life body is one that culturally symbolises relaxation, enjoyment, control, pleasure and wellbeing, at least until a point where the physical processing of alcohol in the (ageing) body asserts a changed relationship. Any attempt to change drinking behaviour will need to take such cultural significance and lived past and present experience into account.
